# Astrocytic APOE4 removal confers cerebrovascular protection despite increased cerebral amyloid angiopathy

**DOI:** 10.1186/s13024-023-00610-x

**Published:** 2023-03-16

**Authors:** Monica Xiong, Chao Wang, Maud Gratuze, Fareeha Saadi, Xin Bao, Megan E. Bosch, Choonghee Lee, Hong Jiang, Javier Remolina Serrano, Ernesto R. Gonzales, Michal Kipnis, David M. Holtzman

**Affiliations:** 1 Department of Neurology, Hope Center for Neurological Disorders, Knight Alzheimer’s Disease Research Center, Washington University School of Medicine, St. Louis, MO 63110 USA; 2 Division of Biology and Biomedical Sciences (DBBS), Washington University School of Medicine, St. Louis, MO 63110 USA; 3 Present Address: Genentech, 1 DNA Way, South San Francisco, CA 94080 USA; 4 Institute for Brain Science and Disease, Chongqing Medical University, Chongqing, 400016 China; 5 Present address: Institute of Neurophysiopathology (INP UMR7051), CNRS, Aix-Marseille Université, Marseille, 13005 France

**Keywords:** APOE, Cerebral amyloid angiopathy, Cerebrovasculature, Astrocyte, Amyloid-β

## Abstract

**Background:**

Alzheimer Disease (AD) and cerebral amyloid angiopathy (CAA) are both characterized by amyloid-β (Aβ) accumulation in the brain, although Aβ deposits mostly in the brain parenchyma in AD and in the cerebrovasculature in CAA. The presence of CAA can exacerbate clinical outcomes of AD patients by promoting spontaneous intracerebral hemorrhage and ischemia leading to CAA-associated cognitive decline. Genetically, AD and CAA share the ε4 allele of the apolipoprotein E (*APOE*) gene as the strongest genetic risk factor. Although tremendous efforts have focused on uncovering the role of *APOE4* on parenchymal plaque pathogenesis in AD, mechanistic studies investigating the role of *APOE4* on CAA are still lacking. Here, we addressed whether abolishing APOE4 generated by astrocytes, the major producers of APOE, is sufficient to ameliorate CAA and CAA-associated vessel damage.

**Methods:**

We generated transgenic mice that deposited both CAA and plaques in which APOE4 expression can be selectively suppressed in astrocytes. At 2-months-of-age, a timepoint preceding CAA and plaque formation, APOE4 was removed from astrocytes of 5XFAD *APOE4* knock-in mice. Mice were assessed at 10-months-of-age for Aβ plaque and CAA pathology, gliosis, and vascular integrity.

**Results:**

Reducing the levels of APOE4 in astrocytes shifted the deposition of fibrillar Aβ from the brain parenchyma to the cerebrovasculature. However, despite increased CAA, astrocytic APOE4 removal reduced overall Aβ-mediated gliosis and also led to increased cerebrovascular integrity and function in vessels containing CAA.

**Conclusion:**

In a mouse model of CAA, the reduction of  APOE4 derived specifically from astrocytes, despite increased fibrillar Aβ deposition in the vasculature, is sufficient to reduce Aβ-mediated gliosis and cerebrovascular dysfunction.

**Supplementary Information:**

The online version contains supplementary material available at 10.1186/s13024-023-00610-x.

## Background

Cerebral amyloid angiopathy (CAA) and Alzheimer Disease (AD) are clinically distinct but share overlapping molecular and genetic features. For example, the earliest detectable pathological marker of both neurodegenerative diseases includes the accumulation of amyloid-β (Aβ). Aβ deposits in the cerebrovasculature as CAA and as neuritic plaques in AD, although CAA co-occurs in 85–95% of AD postmortem brains [1]. The clinical manifestations, however, are disparate: hyper-phosphorylated aggregated forms of tau are linked with cortical atrophy that strongly correlates with cognitive performance in AD [2, 3], whereas CAA-associated bleeds and ischemia give rise to vessel dysfunction [4] that accelerates cognitive impairment in AD and non-AD cases [5–7]. In addition to increasing the risks of intracerebral hemorrhages, vascular Aβ toxicity compromises the neurovascular unit [8] by rendering vascular cells irresponsive to physiological events [9] and impairing perivascular drainage [10], all of which may exacerbate the progression of AD.

Despite different mechanisms by which CAA and AD exacerbate Aβ-mediated pathology, CAA and AD share a significant genetic risk factor that increases the prevalence and severity of both diseases. Apolipoprotein E (APOE) plays a critical role in lipid transport [11], although the ε4 allele of the *APOE* gene is detrimental for both CAA [5, 12–15] and AD [16, 17] by pathogenically enhancing Aβ aggregation and impairing Aβ clearance [18, 19]. This impaired clearance promotes a self-reinforcing cycle that further deposits Aβ along vessels and in the parenchyma to worsen AD and CAA [10, 20, 21]. Additionally, APOE is a major constituent in both CAA [22] and plaques [22–24]. APOE is required for the development of CAA and is a key contributor to fibrillar parenchymal plaque formation [25, 26]. *APOE4* also exhibits Aβ-dependent and -independent effects [27] on the cerebrovasculature by reducing cerebral blood flow [28, 29] and increasing the permeability of the blood–brain barrier (BBB) [30–32]. Although there is certainly a relationship between APOE and CAA on cerebrovascular dysfunction, the underlying mechanisms remain unclear.

In the central nervous system (CNS), astrocytes are the predominant producers of APOE, although reactive microglia [33, 34], injured neurons [35], and certain vascular mural cells [36, 37] also generate APOE. *APOE4*-expressing astrocytes, with or without injury from diseases such as CAA, undergo extensive transcriptional, morphological, and functional remodeling into a pathological state marked by a loss of physiological function [38–46]. Deletion of *APOE4* selectively from astrocytes restores astrocytes and other cells in the CNS to a more “homeostatic” state, which provides protection from disease progression in mouse models of amyloidosis [47, 48] and tauopathy [49]. Both studies in which astrocytic APOE4 was removed detected a decrease in reactive microglia, which is likely associated with an overall decrease in CNS inflammation and, consequently, promotion of neuroprotection. Moreover, pathogenic Aβ or tau were also reduced, although CAA was at very low to undetectable levels in these models and therefore not quantified. In another study, removal of astrocytic APOE4 in otherwise non-transgenic mice was sufficient to provide  certain BBB protection [50]. Given the critical role of astrocytes under basal conditions and the detrimental effects of the ε4 allele of APOE on CAA and the cerebrovasculature, we hypothesized that reduction of APOE4 expression in astrocytes in a CAA mouse model would ameliorate CAA-dependent and/or -independent vascular damage. To address this hypothesis, we utilized inducible Aldh1l1-Cre/ERT2 BAC transgenic mice [51] crossed to 5XFAD (line 7031) mice [52] expressing human APOE4^flox/flox^ [53], a model with extensive CAA, to selectively delete APOE4 expression in astrocytes upon the administration of tamoxifen. In addition to determining whether APOE4 removal from astrocytes protects the cerebrovasculature, we also explored the contributions of astrocytic APOE4 on CAA formation and progression.

## Methods

### Animals

All animal studies conducted abided by institutional animal care and use committee (IACUC) protocols approved by the Animal Studies Committee of Washington University in St. Louis. 5XFAD mice (line Tg7031), a gift from R. Vassar at Northwestern University [52], were crossed for multiple generations to APOE4^flox/flox^ mice [53] to generate 5XFAD APOE4^flox/flox^ mice. Aldh1l1-Cre/ERT2 mice (Jackson Laboratories, Stock No. 031008) were also crossed to APOE4^flox/flox^ mice for several generations to generate Aldh1l1-Cre/ERT2 APOE4^flox/flox^ mice. To generate 5XFAD Aldh1l1-Cre/ERT2 APOE4^flox/flox^ mice, 5XFAD APOE4^flox/flox^ males were crossed to Aldh1l1-Cre/ERT2 APOE4^flox/flox^ females. We refer to mice carrying the 5XFAD and Aldh1l1-Cre/ERT2 genes as “5X+AL+” and their littermates without the Aldh1l1-Cre/ERT2 gene as “5X+AL-”. Littermates without the 5XFAD transgene are designated as “5X-” mice. All mice were housed on a normal 12-h light/dark cycle with free access to food and water. There were no sex differences between males and females and the number of mice used can be found in the figure legends.

### Tamoxifen treatment

Tamoxifen was prepared fresh every month by dissolving in corn oil at 20 mg/mL on a shaker at 37 °C for at least 12 h. At 2-months-of-age, all mice in experimental groups received one daily injection of tamoxifen (200 mg/kg, intraperitoneal) for five consecutive days. Tamoxifen treatment induced cre combination to reduce the expression of APOE4 in astrocytes.

### Tissue harvesting and brain extraction

Mice were first anesthetized with Fatal-Plus (200 mg/kg, intraperitoneal) and then perfused with chilled phosphate-buffered saline (PBS) containing 0.3% heparin. One hemibrain was fixed in cold 4% paraformaldehyde (PFA) for 24 h and then cryoprotected with 30% sucrose at 4 °C for histology. The other hemisphere was dissected into anterior and posterior cortices and flash-frozen for biochemical and gene transcript analyses.

### Histology and imaging quantification

#### Cryosectioning

Hemibrains fixed in 4% PFA were coronally sectioned on a freezing, slicing microtome (Leica) at 30 µm and stored at –20 °C in cryoprotectant solution (0.2 M PBS, 15% sucrose, 33% ethylene glycol).

#### Immunofluorescence

Immunofluorescent staining was conducted as previously described [54]. Briefly, two brains Sects. (180 µm apart) were first permeabilized in Tris-buffered saline (TBS) containing 0.25% Triton-X100 (TBS-X) and then blocked using 3% serum in TBS-X. Brain slices were incubated overnight in primary antibody diluted in 1% serum in TBS-X at 4 °C. Primary antibodies include: biotinylated antibody HJ3.4 for human Aβ_1–13_ (produced in-house, 2 µg/mL), rabbit Aβ_40_ (Thermo Fisher, 44,136, 1:500), rabbit Aβ_42_ (Thermo Fisher, 700,254, 1:500), rabbit APOE antibody (Cell signaling, #13,366, 1:500), biotinylated GFAP for astrocytes (Sigma-Aldrich, MAB3402B, 1:1000), rabbit Iba1 (Wako, 019–19,741, 1:5000), rabbit fibrinogen (Abcam, ab34269, 1:1000), and rat LAMP1 (Developmental Studies Hybridoma Bank, #1D4B, 1:400). The following day, sections were stained for 1 h with secondary antibodies (1:1000) against the primary antibodies. X34 dye (Sigma-Aldrich, SML1954, 1:5000) was used to label amyloid plaques and CAA. For X34 staining, free-floating brain sections were washed (3 X 5 min) and permeabilized in TBS-X for 30 min. Brain tissues were then incubated with X34 working solution (X34 was added at a 1:5000 dilution to 40% ethanol in TBS buffer, pH was adjusted with NaOH at 1:500 dilution). After 20 min incubation, sections were de-stained with X34 washing buffer (40% ethanol in TBS, 3 X 2 min) and then washed with TBS (3 X 5 min). If primary antibodies were used, the sections were subjected to a serum blocking step as mentioned above. For Aβ_40_ and Aβ_42_ staining, sections were first incubated in 88% formic acid prior to staining.

#### Fluorescence imaging and analysis

Immunofluorescence imaging on fixed brain tissue sections was performed at 10X on the Biotek Cytation 5 Imaging Reader (Figs. 2a, f, 4a, f, 5a (left panels), 6a), 20X on the Leica Thunder Imager DMi8 (Figs. 3e, m, S3a, S5a), or 40X oil objective on the Leica Stellaris 5 Confocal microscope at 1024 × 1024 pixel resolution (Figs. 4c, h, 5a (right panels), S4a). Images were analyzed on Fiji software (ImageJ) 1.52v. For area coverage by fluorescent staining, a single threshold was set for all images to determine percent area coverage in the cortex overlying the hippocampus. CAA and vascular Aβ were differentiated from parenchymal plaques based on morphology. CAA in this model appears as transverse band-like deposition of amyloid onto structures that have been morphologically determined to be pial or penetrating blood vessels. The cross-section of CAA has a thin, ring-like appearance where the center is hollow. Parenchymal plaques are generally spherical and often compact. To validate that this assessment of CAA and plaques by morphology was accurate, we co-stained 5XFAD APOE4^flox/flox^ mice for X34 and CD31 for endothelial cells. We confirmed that what we were identifying as X34^+^ CAA vessels were CD31^+^ while X34^+^ parenchymal plaques were CD31^−^, thereby validating that we can distinguish CAA vessels from parenchymal plaques with high confidence. For colocalization analysis, colocalization of APOE with microglia or astrocytes was performed using the built-in image calculator AND operator. The percent of colocalization between the two channels (APOE and microglia, APOE and astrocytes) was normalized to the plaque or CAA area. Colocalization analysis of individual CAA vessels was conducted mostly in ~ 20 µm vessels. To quantify the number of astrocytes or microglia surrounding CAA or plaques, images containing only CAA or fibrillar plaque were taken from cortical regions overlying the hippocampus of *n* = 8–10 mice per group. The number of astrocytes or microglia surrounding CAA or plaque were manually counted and normalized to the CAA or plaque size. Images containing mixed CAA/plaque pathology where CAA and plaques were co-deposited were not used because CAA- or plaque-specific effects could not be distinguished. For quantification of LAMP1 around plaques or CAA, we determined the percent area covered by LAMP1 around individual plaques or CAA vessels and normalized these values to the percent area covered by X34^+^ plaques or CAA, respectively. For this analysis, we assessed six CAA vessels or plaques per mouse and averaged these values for one biological replicate. Images were processed and analyzed while blind to experimental conditions.

#### Microhemorrhage analysis

Staining for hemosiderin deposits was performed as previously described with minor modifications [54]. Eight brain sections spaced 180 µm apart were incubated in 2% potassium ferrocyanide (P3289; Sigma-Aldrich) in 2% hydrochloric acid for 30 min. Brain sections were imaged with Nanozoomer 2.0-HT slide scanner at 40 × magnification. Quantifications of the size and number of microhemorrhages was performed by manually tracing hemosiderin + deposits using NDP.View2 software. Analyses and tracings were completed by investigators blind to the treatment.

### Live imaging of pial arteries

Live vascular imaging for cerebrovascular function was performed as previously described with minor modifications [54]. To sample for blood gases, one femoral artery was cannulated (Nova Biomedical, BioProfile pHOx Analyzer) under isoflurane (4% induction, 1.5% maintenance) anesthesia. A tracheostomy was performed for mechanical ventilation (Harvard Apparatus, MiniVent Ventilator) supplemented with 0.5% flow of 100% O_2_. To visualize leptomeningeal vessels under a fluorescent microscope, fluorescein-dextran (Life Technologies, D1823, 150 uL at 12.5 mg/mL) was injected retro-orbitally. Isoflurane is a vasodilator, therefore mice were weaned off of isoflurane and instead anesthetized with pentobarbital (i.p., 1.35 mL/kg from 50 mg/mL stock solution for first dose, 0.70 mL/kg for subsequent doses) during live imaging. Next, a craniotomy was performed by securing mice to a custom-built stereotaxic device (Instrument Machine Shop at Washington University School of Medicine) and removing the right parietal bone (4 mm in diameter). The window was bathed in artificial cerebrospinal fluid (aCSF, in mM: 125 NaCl, 26 NaHCO3, 1.25 NaH2PO4, 2.5 KCl, 1 MgCl2, 1 CaCl2, and 25 mM glucose). CAA on pial vessels was labeled with X34 dye. Pial arteries were imaged using the Nikon Eclipse 600ME digital video microscopy system (Nikon Instruments Inc.) at 20 × with water-immersion lenses at 1024 × 1024 pixels using MetaMorph imaging software version 7.10.2 (Molecular Devices). The vascular smooth muscle cell − dependent vasodilator *S*-Nitroso-*N*-acetyl-penicillamine (SNAP; Sigma-Aldrich, N3398, 50 μM) was topically applied to the window for 5 min. Images were analyzed with ObjectJ software plug-in version 1.04q in Fiji (ImageJ) and the diameters of 6–8 ~ 30 µm vessel segments in length were measured. Data were calculated as percent vasodilatory change from baseline. Vessel segments from one vessel were averaged and analyzed as an individual biological replicate. Data were generated from 9 vessels in 4 mice (5X+AL-) and 6 vessels in 3 mice (5X+AL+), with no more than two vessels from one mouse.

### Protein extraction and sandwich ELISA

The protocol for tissue lysates extraction and detection of human Aβ *(*55) or APOE [49] was previously described in detail. Mouse cortical tissue samples (~ 15 mg) were sequentially homogenized with pre-chilled PBS and 5 M guanidine buffer in the presence of protease inhibitor (Roche, 11,697,498,001) and phosSTOP inhibitor (Roche, 04,906,845,001). After adding magnetic beads (Next Advance, ZrOB05) and 20 µl PBS/1 mg tissue, tissue were homogenized for 45 s on a bead homogenizer (Next Advance, Bullet Blender Strom 24) on setting 3. Then, homogenates were centrifuged for 30 min at 15,000 rpm at 4 °C. The supernatant was collected as the PBS salt-soluble fraction. Next, the same volume of guanidine buffer was added to the pellet and homogenized again on setting 8 for 3 min. The homogenates were rotated for 1 h at room temperature, followed by centrifugation for 30 min at 15,000 rpm at 4 °C. Finally, the supernatant was collected as the guanidine-soluble (“insoluble”) fraction. Both fractions were stored at -80 °C until further use. The levels of Aβ_40_, Aβ_42_, and APOE in PBS and 5 M guanidine fractions were measured by sandwich ELISA and normalized to the tissue weight. For Aβ_40_, anti-Aβ_35-40_ HJ2 (in-house) was used as the capture antibody and biotinylated anti-Aβ_13-18_ HJ5.1 (in-house) was used as the detection antibody. For Aβ_42_, anti-Aβ_37-42_ HJ7.4 (in-house, mouse monoclonal) was the capture antibody and biotinylated anti-Aβ_13-18_ HJ5.1 (in-house, mouse monoclonal) was the detection antibody. The coating antibody for human APOE was HJ15.3 (in-house, mouse monoclonal), and the capture antibody was HJ15.7-biotinylated (in-house, mouse monoclonal) [53].

### Astrocyte morphology analysis

Immunofluorescent sections containing X34 and GFAP were imaged on the Leica Stellaris 5 confocal microscope (40 × oil objective, 1024 × 1024 pixel resolution). Images exclusively containing CAA or fibrillar plaques (no mixture of plaque/CAA pathology) from cortical regions overlying the hippocampus were used for this analysis. Simple Neurite Trace (SNT; ImageJ plug-in open-source tool) was used to reconstruct two-dimensional arbors of GFAP^+^ astrocytic processes to generate morphological readouts, including size (convex hull), number of processes, total sum of processes length, number of processes points, and mean process length. Four to five astrocytes were randomly chosen from two sections in each mouse and did not have processes that touched another cell or were truncated.

### Fluidigm qPCR

RNA was extracted from frozen anterior cortical tissue with the RNeasy Mini Kit (Qiagen) and converted to cDNA using the RNA-to-cDNA kit per the manufacturer’s instructions. In collaboration with Genome Technology Access Core at Washington University, gene expression was performed with Fluidigm Biomark HD Real-Time PCR System using Taqman primers. Relative gene expression was normalized to *Actb*.

### Statistical analysis

GraphPad Prism 9.5.0 was used for statistical analyses. As stated in the figure legends, data are presented as mean ± standard deviation (SD) for group sizes less than 10; otherwise, data are presented as mean ± standard error of the mean (SEM). No other statistical comparisons were significant unless otherwise noted. Statistical significance between two groups was calculated using an unpaired student’s *t*-test (two-tailed). If data had unequal variances, a *t*-test with Welch’s correction was used.

## Results

### Removal of astrocytic APOE4 redistributes Aβ from the brain parenchyma to the cerebrovasculature

**Fig. 1:**
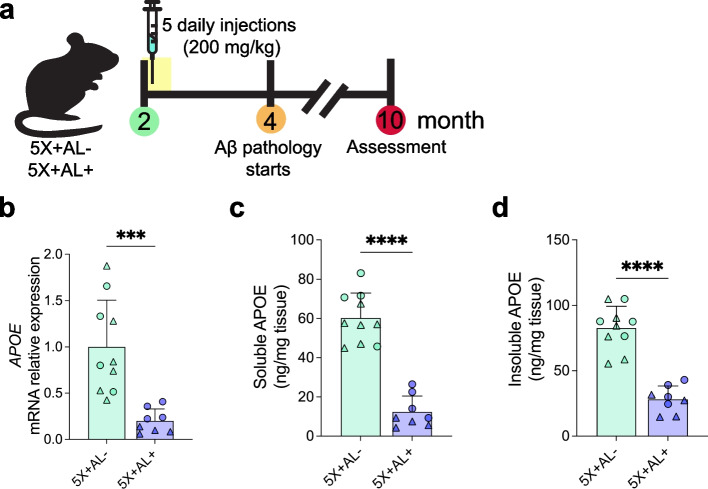
Tamoxifen-induced Cre recombination reduces both APOE mRNA and protein. **a** Schematic timeline of tamoxifen treatment (5 daily injections at 200 mg/kg) in 2-month-old 5XFAD (line 7031) x *APOE4*^flox/flox^ (5X+AL-) or 5XFAD (line 7031) x *APOE4*^flox/flox^ x Aldhl1l-Cre/ERT2 (5X+AL+) mice, assessed at 10-months of age. **b** Relative expression of *APOE* mRNA normalized to beta-actin in cortex. **c**, **d** PBS-soluble and guanidine-HCL-soluble (“insoluble”) APOE protein concentrations assessed by ELISA from cortex. Data expressed as mean ± SD, student’s *t*-test (two-sided) performed for all statistical analyses except (**b**), where Welch’s *t*-test was performed. ∆ = males, ○ = females. ***P < 0.001, ****P < 0.0001. No other statistical comparisons are significant unless indicated

To achieve astrocyte-specific deletion of *APOE4* in a mouse model with mixed CAA and plaque pathology, we generated 5XFAD (line 7031) APOE4^flox/flox^ transgenic mice either with (5X+AL+) or without (5X+AL-) the Aldhl1l-Cre/ERT2 gene [51–53]. Cre-mediated recombination to eliminate expression of APOE4 from astrocytes was achieved via five consecutive days of intraperitoneal (i.p.) tamoxifen treatment in 2-month-old 5X+AL+ or 5X+AL- mice, a timepoint preceding plaque/CAA development (Fig. 1a). Tamoxifen treatment resulted in robust reduction of cortical *APOE4* mRNA (Fig. 1b) and protein (Fig. 1c, d) in 10-month-old 5X+AL+ mice. There was no sex effect on *APOE4* mRNA (Figure S1a) and protein levels (Figure S1b, c) before or after tamoxifen administration.

**Fig. 2:**
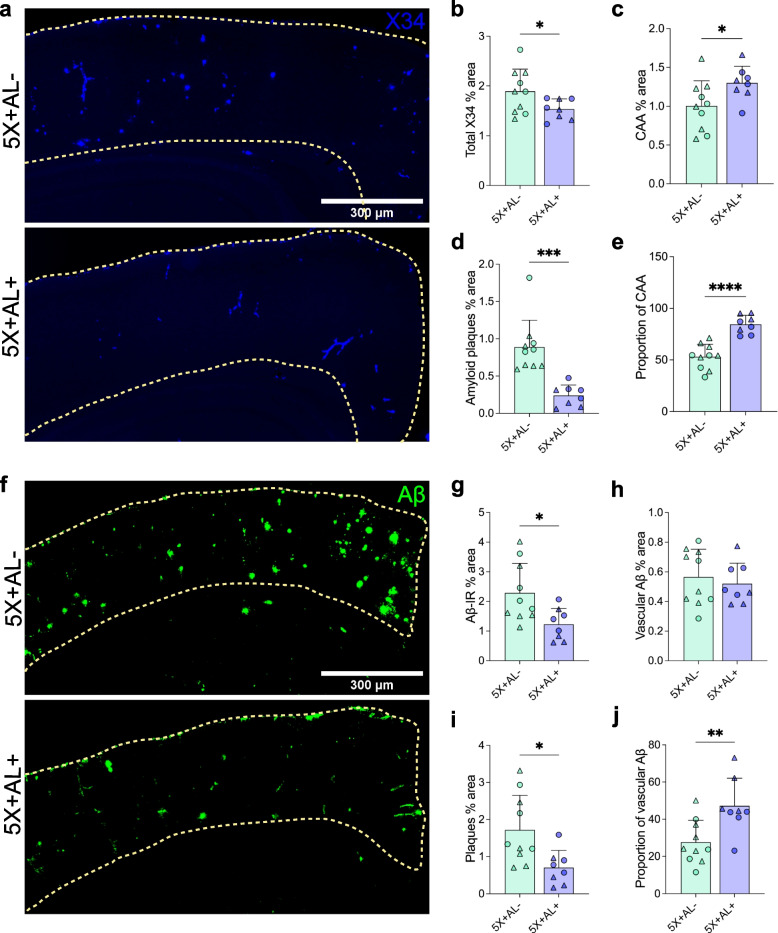
Removal of astrocytic APOE4 before amyloid deposition shifts Aβ distribution from plaques to the cerebrovasculature. **a**–**d**, X34 staining for fibrillar plaques/CAA (**a**) with percent area coverage of total X34 (**b**), CAA (**c**), and amyloid plaques (**d**) in the cortex overlying the hippocampus of 10-month-old 5X+AL- or 5X+AL+ mice after astrocytic APOE4 removal at 2-months-of-age. **e**, Proportion of CAA in total X34^+^ staining. **f–i**, Aβ immunoreactivity (Aβ-IR) (**f**) with percent area coverage of total Aβ-IR (**g**), vascular Aβ-IR (**h**), and plaques (**i**) in the cortex overlying the hippocampus. **j** Proportion of vascular Aβ in total Aβ-IR. Scalebar: 300 µm. Data expressed as mean ± SD, unpaired student’s *t*-test (two-sided) performed for all statistical analyses except (**b**), where Welch’s *t*-test was performed. ∆ = males, ○ = females. *P < 0.05, **P < 0.01, ***P < 0.001, ****P < 0.0001. No other statistical comparisons are significant unless indicated

Previous studies have demonstrated that *APOE4* facilitates CAA development whereas global *APOE* deficiency in mice prevents CAA pathology [25]. Therefore, we hypothesized that the removal of astrocytic APOE4 would reduce both CAA and Aβ plaque burden in 10-month-old 5X+AL+ mice. Indeed, we detected a reduction of total fibrillar Aβ deposits and fibrillar plaques (Fig. 2a, b, d), as well as total Aβ immunoreactivity (Fig. 2f, g) and Aβ^+^ plaques (Fig. 2i) in cortical regions overlying the hippocampus. However, to our surprise, reducing APOE4 from astrocytes resulted in increased fibrillar Aβ in CAA (Fig. 2c, e). Although vascular Aβ mostly deposits in a fibrillar form, there is also a component that is Aβ^+^ immunoreactive and not fibrillar. Aβ^+^ staining was detected in vessels but this staining was not altered by astrocytic APOE4 deficiency (Fig. 2h). However, the proportion of Aβ^+^ staining in the vasculature increased (Fig. 2j), suggesting a greater propensity for Aβ to accumulate in vessels after APOE4 removal from astrocytes. Certain mouse models of amyloidosis reveal a sex effect on Aβ plaque accumulation, although, in our study, we observed no sex-dependent difference in the coverage of cortical X34^+^ fibrillar load or Aβ^+^ immunoreactivity in vessels or plaques (Figure S2). In addition to amyloid load in the cortex, we also assessed amyloid pathology in a brain region with dense plaque/CAA burden, the subiculum (Figure S3a). Although there was no change in total fibrillar plaque/CAA deposition in the dorsal subiculum after astrocytic APOE4 removal (Figure S3b), we again observed a two-fold increase in CAA (Figure S3c) and a two-fold decrease in plaque coverage (Figure S3d). These results suggest that whereas complete embryonic ablation of APOE from all cell types protects from CAA progression [25], selective astrocytic APOE4 deletion in adult mice beginning at 2-months-of-age resulted in exacerbated CAA in 10-month-old 5X+AL+ mice.

**Fig. 3:**
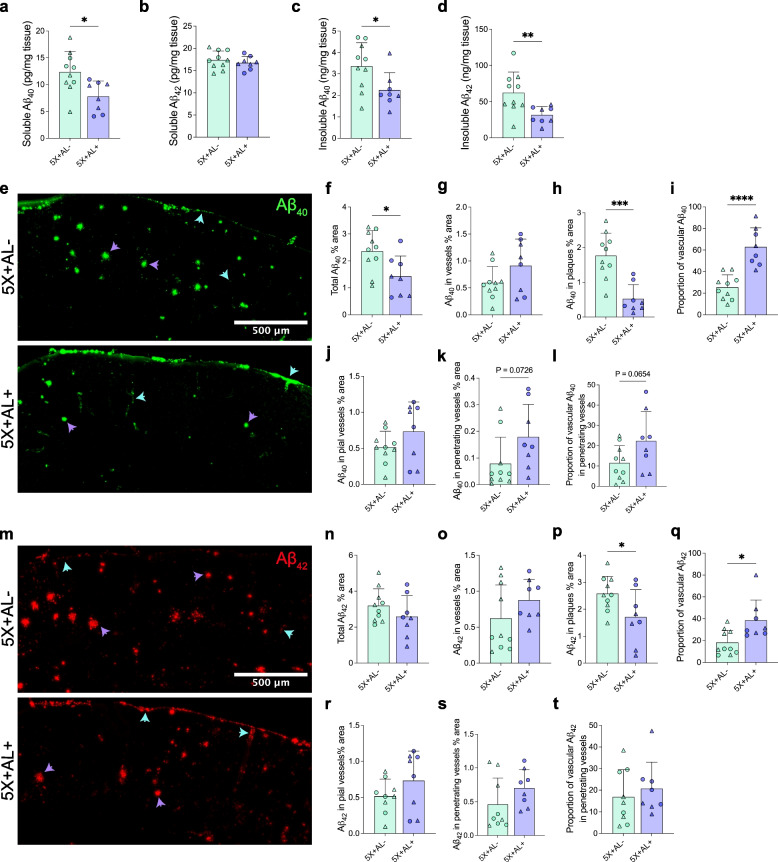
Astrocytic APOE4 regulates the distribution of Aβ_40_ and Aβ_42_ deposition in plaques, parenchymal vessels, and leptomeningeal vessels. **a**–**d**, PBS-soluble and guanidine-HCL-soluble (“insoluble”) Aβ_40_ and Aβ_42_ protein concentrations assessed by ELISA from cortex. **e**–**h** Aβ_40_ immunoreactivity (IR) (**e**) with percent area coverage of total Aβ_40_ (**f**), total vascular Aβ_40_ (**g**), and Aβ_40_ in plaques (**h**). **i** Proportion of vascular Aβ_40_ in total Aβ_40_ IR. **j**, **k** Aβ_40_ IR restricted to pial vessels (**j**) and penetrating vessels (**k**). (**l**) Proportion of vascular Aβ_40_ in penetrating vessels out of total vascular Aβ_40_. **m**–**p**, Aβ_42_ immunoreactivity (IR) (**m**) with percent area coverage of total Aβ_42_ (**n**), total vascular Aβ_42_ (**o**), and Aβ_42_ in plaques (**p**). **q** Proportion of vascular Aβ_42_ in total Aβ_42_ IR. **r, s**, Aβ_42_ IR restricted to pial vessels (**r**) and penetrating vessels (**s**). **t** Proportion of vascular Aβ_42_ in penetrating vessels out of total vascular Aβ_42_. Blue arrow = vascular Aβ. Purple arrows = plaques. Scalebar: 500 µm. Data expressed as mean ± SD, student’s *t*-test (two-sided) performed for all statistical analyses except (**d**), where Welch’s *t*-test was performed. ∆ = males, ○ = females. *P < 0.05, *P < 0.01, ***P < 0.001, ****P < 0.0001. No other statistical comparisons are significant unless indicated

The increase in CAA after astrocytic APOE4 removal prompted us to ask whether alterations in Aβ_40_ versus Aβ_42_ composition in the brain could explain the shift in higher vascular amyloid burden. Aβ_40_ has a greater propensity to accumulate along the vasculature, although in vivo and in vitro studies reveal that Aβ_42_ is necessary for the initial seeding of vascular Aβ deposition [21, 56]. Therefore, an increase in CAA may be reflected by higher concentrations of Aβ_40_ that have accumulated in the vasculature. To address this hypothesis, we performed bulk ELISA on cortical tissue for PBS-soluble and 5 M guanidine-HCl-soluble (“insoluble”) Aβ_40_ and Aβ_42_ (Fig. 3a–d). As expected, we detected a reduction of insoluble Aβ_42_, which is the main species of Aβ in plaques (Fig. 3d). However, there was also a decrease in soluble Aβ_40_ (Fig. 3a) and insoluble Aβ_40_ protein concentrations (Fig. 3c) after astrocytic APOE4 removal despite elevated CAA by histology (Fig. 2c). One caveat is that bulk ELISA does not allow for distinction between Aβ_40_ in the vasculature versus plaque, and it is possible that Aβ_40_ also deposits abundantly in plaques in this mouse model; the lowering of overall Aβ_40_ by ELISA in mice without astrocytic APOE4 may therefore instead be a reflection of plaque reduction. Thus, we also measured the percent area coverage of Aβ_40_ and Aβ_42_ localized to either plaques or vessels via immunostaining (Fig. 3e–t). In mice lacking astrocytic APOE4, there was a decrease in total Aβ_40_ **(**Fig. 3f) and Aβ_40_ in plaques (Fig. 3h), but not in vascular Aβ_40_ (Fig. 3g). However, there was a shift in the proportion of Aβ_40_ depositing in vessels in mice with astrocytic APOE4 (~ 20%) compared to those without (~ 60%), suggesting that without astrocytic APOE4, Aβ_40_ peptides aggregated at comparable levels in the vessels but were possibly cleared or degraded more quickly in the brain parenchyma (Fig. 3i). This is further supported by our ELISA data which revealed a decrease in soluble Aβ_40_ but not Aβ_42_ (Fig. 3a, b), possibly as a result of more rapid Aβ_40_ clearance. We further parsed out the regional deposition of Aβ_40_ by assessing its coverage in leptomeningeal vessels along the surface of the brain compared to penetrating vessels in the brain parenchyma. There was no change in pial accumulation of Aβ_40_ (Fig. 3j); however, there was a two-fold increase in Aβ_40_ depositing in the penetrating vessels of the brain parenchyma (Fig. 3k, *P*=0.073; Fig. 3l, *P*=0.065). This suggests that after neurons release Aβ_40_ into the brain parenchyma, there is a pool of Aβ_40_ that does not aggregate with plaques. Instead, Aβ_40_ peptides may be shuttled out via the interstitial fluid and perivascular drainage pathway along parenchymal arteries where they aggregate or exit through the pial arteries/CSF. Interestingly, there is no significant reduction of total Aβ_42_ (soluble or insoluble forms are indistinguishable by histology; Fig. 3m, n), although we detected a decrease in Aβ_42_ accumulating in plaques (Fig. 3p). Further analysis demonstrated a lack of difference between pial versus penetrating vascular Aβ_42_ accumulation (Fig. 3r–t), supporting previous findings that Aβ_40_ preferentially aggregates around vessels and may contribute to greater CAA accumulation in mice lacking astrocytic APOE4.

### Depletion of APOE4 in astrocytes alters disease-associated glial signatures

Cre-mediated recombination to remove astrocytic APOE4 in our mouse model reduced overall APOE mRNA and protein levels. To assess the magnitude of APOE4 expression change at the cellular level, we investigated the spatial distribution and expression of APOE4 in two major cell types that upregulate a disease-associated transcriptional profile in response to Aβ pathology [57, 58]: astrocytes and microglia. As expected, without Cre recombination (5X+AL- mice), APOE was mostly localized in GFAP^+^ astrocytes and IBA1^+^ microglia surrounding CAA or plaques; however, there was also APOE expression in astrocytes in cortical regions devoid of amyloid deposition but not in microglia (Figure S4a). Deletion of *APOE4* from astrocytes resulted in an overall decrease of APOE4 protein in cortical regions with or without CAA/plaque pathology (Figure S4a, b). The reduction of APOE was exclusive to GFAP^+^ astrocytes (Figure S4c) and not observed in IBA1^+^ microglia (Figure S4d), indicating that APOE4 expression in microglia was not altered. Altogether, our results suggest that we achieved robust APOE4 protein reduction specific to astrocytes.

**Fig. 4:**
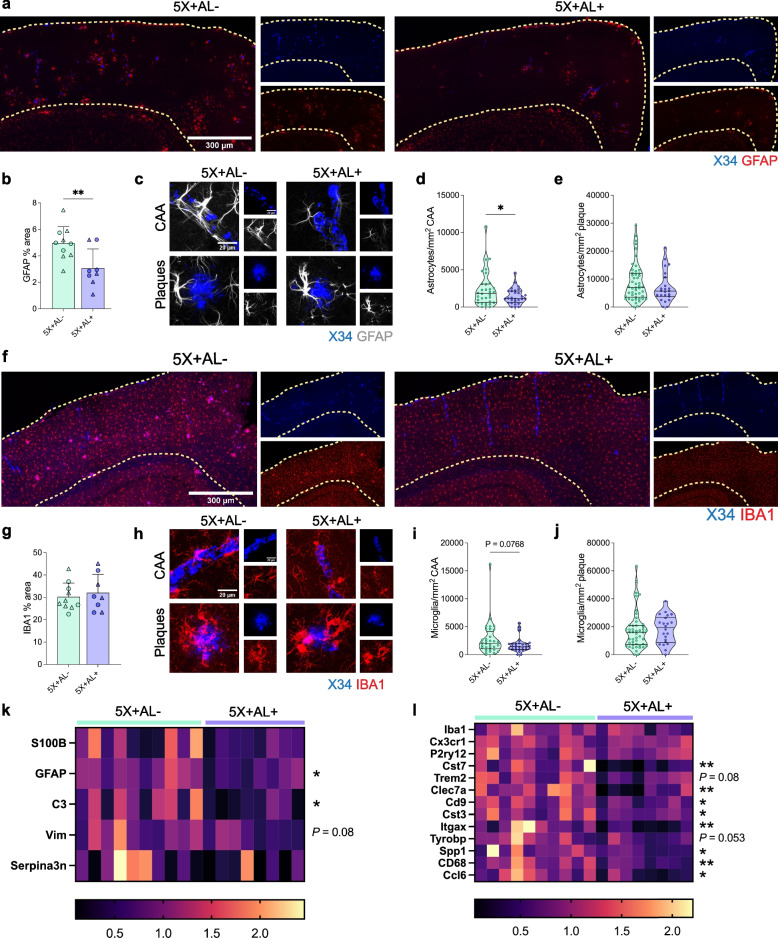
Depletion of APOE in astrocytes diminishes certain disease-associated glial signatures. **a**–**e**, GFAP^+^ astrocytic staining (**a**) and quantification of area coverage (**b**) in the cortex of 10-month-old 5X+AL- or 5X+AL+ mice. Scalebar: 300 µm. Representative images (**c**) and quantification of the number of astrocytes surrounding CAA (**d**) or plaques (**e**). Scalebar: 20 µm. Each point represents the number of astrocytes surrounding a single CAA or plaque normalized to that CAA or plaque area from n = 8–10 mice per group. **f–j**, IBA1^+^ microglial coverage (**f**) and quantification of area coverage (**g**) in the cortex overlying the hippocampus. Scalebar: 300 µm. Per plaque analysis of number of microglia clustering CAA (**h**, **i**) or plaques (**j**). Scalebar: 20 µm. Each point represents the number of microglia surrounding a single CAA or plaque normalized to that CAA or plaque area from n = 8–10 mice per group. **k, l**, Relative mRNA expression of homeostatic and disease-associated astrocytic (**k**) and microglial genes (**l**). Data imputed for *Clec7a* mRNA (column 10) but not used in statistical analysis. Each column represents an individual mouse. Data expressed as mean ± SD, student’s *t*-test (two-sided) performed for all statistical analyses except (**d**), (**e**), (**k** – *S100β*), and (**l** – *Cst7*, *Cst3*, *Itgax*, *Spp1*) where Welch’s *t*-test was performed. ∆ = males, ○ = females. *P < 0.05, **P < 0.01. No other statistical comparisons are significant unless indicated

Next, to evaluate the effects of APOE4 removal on Aβ-mediated gliosis, we assessed the expression pattern of astrocytes and microglia in the cortex. Staining for GFAP^+^ astrocytes revealed an overall reduction of cortical GFAP^+^ astrocytic reactivity in the absence of astrocytic expression of *APOE4* (Fig. 4a, b). In the dorsal subiculum, a region without total amyloid reduction (Figure S3), there was also no change in GFAP area coverage (Figure S5), initially suggesting that amyloid, regardless of the compartment it deposits in, drives GFAP^+^ reactive astrocytosis. However, co-analysis of GFAP^+^ astrocytes with X34^+^ amyloid plaques or CAA revealed that although there was a strong response of GFAP^+^ astrocytes surrounding remaining plaques in astrocytes without APOE4, there was less GFAP^+^ astrocyte reactivity engaging CAA (Fig. 4c–e). Although APOE4 was markedly reduced in astrocytes around both plaques and CAA, the percent area covered by GFAP^+^ astrocytes surrounding plaques was maintained regardless of APOE4 expression. This suggests that once amyloid plaques form, astrocytic reactivity can be maintained in an APOE-independent manner. Additionally, reactive GFAP^+^ astrocytes may undergo distinct morphological remodeling, such that astrocytes in a more “homeostatic” state may display highly ramified processes, whereas “reactive” astrocytes have more retracted processes and hypertrophy, depending on the insult or injury. To differentiate between the astrocytes with or without APOE4 surrounding CAA versus plaques, we assessed the morphology of GFAP-reactive astrocytes. Overall, APOE4 removal from astrocytes did not modify the size or complexity of their morphology (Figure S6a–f). Astrocytes that engage plaques versus CAA, however, demonstrate altered morphology: astrocytes that contact plaques had a greater number (Figure S6c) and length of processes (Figure S6d), marked by increased arborization (Figure S6e). However, on average, these processes were shorter (Figure S6f), representative of numerous retracted terminal processes and implying that amyloid plaques may induce subtle differences such as greater toxicity than CAA to associated astrocytes. Combined, these findings suggest that fewer GFAP^+^ astrocytes were associated with CAA after astrocytic APOE4 removal and that they adopted a morphological state that was possibly less reactive than those contacting the few plaques that remain. More readouts, including functional, are necessary to understand the complexity of these astrocytes surrounding plaques/CAA.

Given that there was an overall dampened GFAP^+^ astrocytic response following astrocyte *APOE4* removal, we were interested in whether astrocyte-microglia crosstalk was affected. To address this question, we assessed the microglial response with IBA1 staining. We detected no changes in IBA1^+^ microglia in the cortex or at the per plaque/CAA level by immunohistochemistry (Fig. 4f–j). When we probed for specific disease-associated glial genes using quantitative PCR (qPCR), we detected reductions in reactive astrocytic gene expression including in *Gfap* and *C3* (Fig. 4k) in the cortex. Interestingly, we also detected reductions in disease-associated microglia (DAM)/microglial neurodegenerative disease (MGnD) genes such as *Cst7*, *Clec7a*, and *Spp1* (Fig. 4l), suggesting that microglia adopted a dampened DAM/MGnD state despite no changes in IBA1^+^ microglia staining. In summary, these data suggest that despite the elevation of CAA in the brain following removal of astrocyte APOE4, there was a reduction of several disease-associated microglial and astrocytic genes. This suggests a modified glial state, possibly due to altered Aβ species and conformation that may be protective in the brain and cerebrovasculature and which we sought to confirm in follow-up functional assays.

### Astrocytic APOE4 deficiency dampens neurodegeneration and cerebrovascular damage

**Fig. 5:**
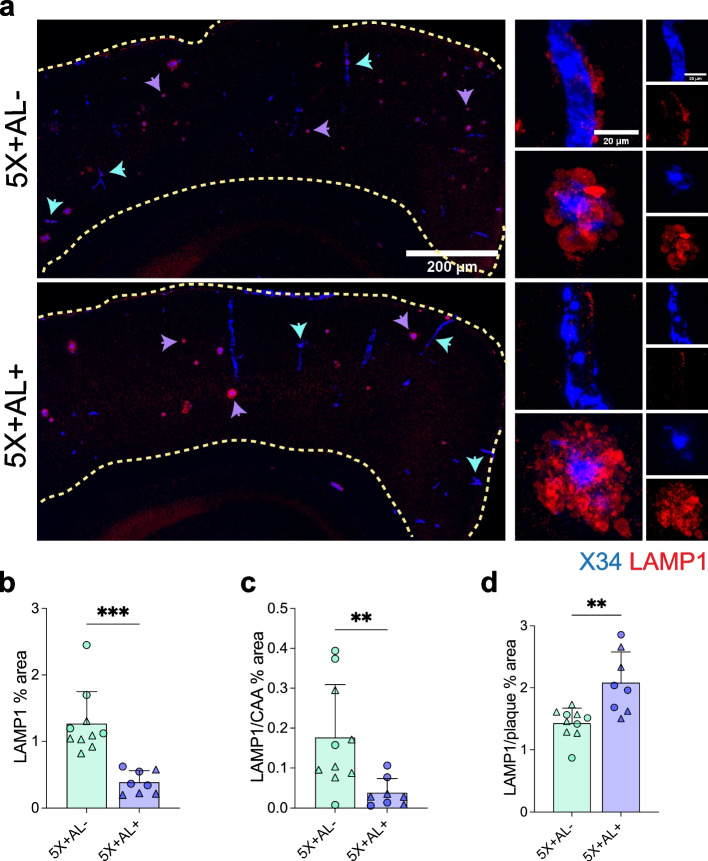
Astrocytic APOE removal protects against dystrophic neurites. a–d, LAMP1 staining for dystrophic neurites (**a**) with quantification of the percentage of total LAMP1 in the cortex (**b**), percent area coverage of LAMP1 staining around CAA normalized to percent area covered by X34^+^ CAA (**c**), and percent area coverage of LAMP1 staining around plaques normalized to percent area covered by X34^+^ plaques (**d**). Scalebar: 200 µm. Purple arrowheads: LAMP1 around plaques. Blue arrowheads: LAMP1 around CAA. Data expressed as mean ± SD, student’s *t*-test (two-sided) performed for all statistical analyses except (**b**) and (**c**), where Welch’s *t*-test was performed. ∆ = males, ○ = females. *P < 0.05, **P < 0.01, ****P < 0.0001. No other statistical comparisons are significant unless indicated

Despite increased CAA, we determined that astrocytic APOE4 removal reduced disease-associated neuroinflammation, an outcome which may provide downstream protection to the CNS. To assess whether APOE4 reduction provides neuroprotection to plaque- and CAA-associated neural processes, we assessed neuritic dystrophy by labeling for LAMP1, a lysosomal marker that is highly enriched in large, swollen axons surrounding amyloid and CAA [59, 60]. In the cortex, the absence of astrocytic APOE4 resulted in significantly reduced LAMP1^+^ dystrophic neurites (Fig. 5a, b). Further analysis revealed that there was a decrease in LAMP1^+^ reactivity around CAA (Fig. 5c), whereas LAMP1 immunoreactivity was increased around remaining parenchymal fibrillar plaques (Fig. 5d), similar to what has been observed in amyloid-depositing APOE knockout mice [61]. Because neuritic dystrophy is generally more prominent around plaques than CAA, the overall reduction of neuritic dystrophy in the cortex of mice without astrocytic APOE4 was likely driven by the striking reduction of total fibrillar plaques in the parenchyma.

**Fig. 6:**
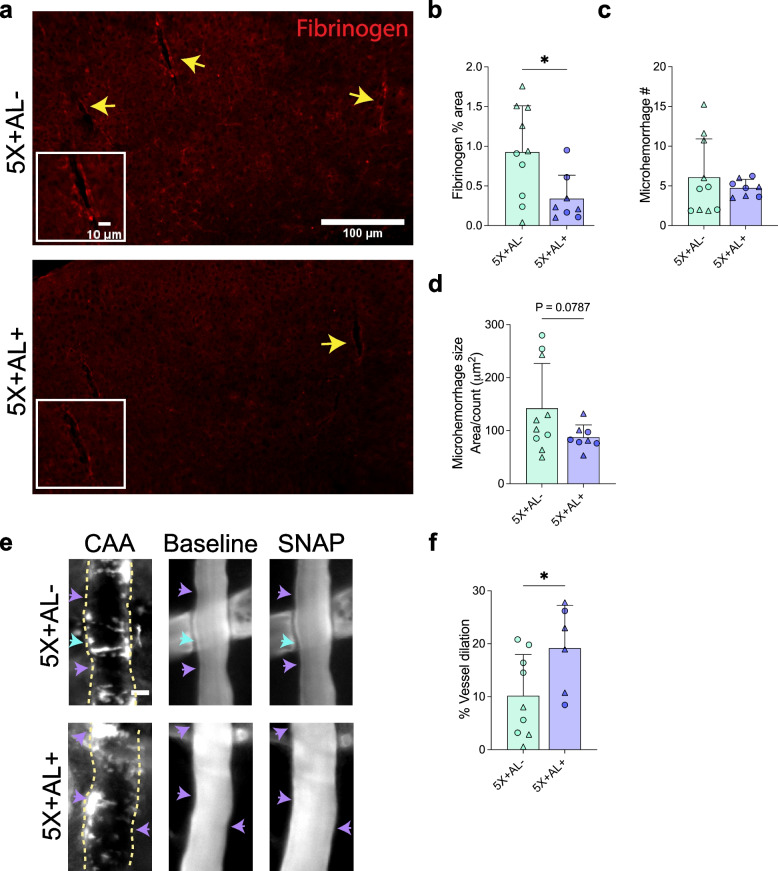
Vascular degeneration is ameliorated despite elevated CAA. **a**, **b** Staining for the blood-derived protein fibrinogen (**a**) with quantification of percent area covered (**b**) in the cortex of 10-month-old 5X+AL- or 5X+AL+ mice. Yellow arrow: fibrinogen staining. Scalebar: 100 µm. **c**, **d**, Average microhemorrhage number (**c**) and size (**d**) via Prussian blue staining. **e**, **f**, In vivo assessment of leptomeningeal function measured by percent vasodilatory change from baseline after topical application of vascular smooth muscle cell-dependent molecule (SNAP). Purple arrowheads: Increased dilation. Blue arrowheads: No change in dilation. Data from 9 vessels in 4 mice (5X+AL-) and 6 vessels in 3 mice (5X+AL+). Scalebar: 15 µm. SNAP = S-Nitroso-N-acetylpenicillamine. Data expressed as mean ± SD, student’s *t*-test (two-sided) performed for all statistical analyses except (**c**) and (**d**), where Welch’s *t*-test was performed. ∆ = males, ○ = females. *P < 0.05, ***P < 0.001. No other statistical comparisons are significant unless indicated

Disruption of cerebrovascular integrity is a hallmark of CAA that can lead to BBB disruption and a loss of physiological vessel function. To determine whether the BBB was compromised in 5X+AL+ mice exhibiting higher CAA load, we stained for the presence of fibrinogen, a blood-derived protein that is normally barred from entering the brain parenchyma (Fig. 6a). Surprisingly, despite the increase in CAA, there was reduced fibrinogen extravasation following removal of astrocyte APOE4 (Fig. 6b). We also investigated whether elevated CAA increased CAA-induced microhemorrhages, but found no differences in the number of microhemorrhages between groups (*P* = 0.07; Fig. 6c, d). The reduction in fibrinogen from control mice prompted us to test whether CAA-laden vessels also exhibit improved vascular function. CAA deposition in the smooth muscle cell (SMC) layer renders arterioles less responsive to molecules that stimulate vasodilation. In live, awake mice, we topically infused a SMC vasodilator (*S*-Nitroso-*N*-acetylpenicillamine, SNAP) that leads to nitric oxide production and measured the vasodilatory response in CAA-containing leptomeningeal vessels on the surface of the brain (Fig. 6e). Relative to 5XFAD mice expressing APOE4, we found that SNAP led to significantly increased vasodilation in 5XFAD mice following removal of astrocyte APOE4 (Fig. 6f). Astrocytic removal therefore provided protection for the BBB and promoted vasoreactivity in SMCs. In summary, our results indicate that the absence of APOE4 from astrocytes resulted in more CAA but fewer parenchymal plaques that reduced Aβ-associated gliosis and improved cerebrovascular function.

## Discussion

The ε4 allele of *APOE* strongly influences risk for AD and CAA. APOE4 impairs and/or competes with Aβ for clearance in addition to accelerating Aβ seeding and fibrillogenesis, which potentially drives the earlier onset of Aβ pathology in *APOE4* carriers with AD. Similarly, *APOE4* carriers with CAA have accelerated prevalence and increased severity of CAA [5, 12], with the additional risk of earlier hemorrhage onset [13]*. *The underlying mechanisms of APOE4 contributions to CAA are not clear. Recently, our lab demonstrated that the removal of APOE4 secreted by astrocytes is sufficient to strongly reduce Aβ parenchymal plaque burden [48] as well as tau-mediated neurodegeneration [49] by potentially altering astrocyte-microglia crosstalk. In this current study, our goal was to investigate the contributions of astrocytic APOE4 to CAA and CAA-associated vascular dysfunction.

Our group has previously shown that genetic ablation of murine APOE is sufficient to prevent the development of CAA in Aβ-depositing mice [25]. However, complete genetic ablation of APOE body wide may not be a viable therapeutic approach given the critical role of both peripheral- and brain-derived APOE in lipid metabolism. Therefore, we asked whether selective removal of APOE from astrocytes, the major producers of APOE in the brain, is sufficient to reduce CAA and CAA-related vessel damage. We hypothesized that lowering the levels of total APOE4 by removing astrocytic APOE4 (~ 75% reduction of total APOE4) would reduce both Aβ plaque and CAA burden in a mouse model of mixed CAA/plaque deposition (5X+AL+). Indeed, Aβ and amyloid plaques were reduced in 5X+AL+ mice. Interestingly, however, there was a concomitant increase in CAA such that Aβ deposition shifted from ~ 50% to ~ 20% in parenchymal plaques and from ~ 50% to ~ 80% in CAA. Similar to previous findings, the remaining parenchymal amyloid plaques in mice without astrocytic APOE4 have a more dispersed rather than dense core morphology [48]. However, to our surprise, the increase in CAA found in the absence of astrocytic APOE4 did not exacerbate CAA-associated damage but rather promoted improved vascular function. Below, we discuss (1) possible explanations for the shift of Aβ from brain parenchyma to the cerebrovasculature, (2) the protective effect of APOE removal on the cerebrovasculature despite increased vascular Aβ, and (3) the implications of our findings on APOE-based therapeutics.

In the brain, APOE may act as a “seed” to self-assemble [62] or directly bind Aβ for fibrillization [63–67]. In fact, in the absence of APOE, transgenic mice that deposit Aβ plaques have enhanced soluble Aβ clearance [68], suggesting that either APOE competes with Aβ for clearance and/or retains Aβ in the brain to reduce clearance. Aβ is cleared from the brain via the perivascular pathway [10], BBB transcytosis [69], cellular uptake [70], and/or interstitial fluid/cerebrospinal fluid (ISF/CSF) bulk flow [71]. APOE is necessary to “seed” Aβ into fibrils at the vasculature for the development of CAA [25]. In our study, we reduced the total protein concentration of APOE4 in the brain by ~ 75%. The remaining APOE4, derived from disease-associated microglia, perivascular macrophages, endothelial cells, pericytes, perivascular fibroblasts, or smooth muscles cells [72], likely contributed to CAA development. Although the contributions of APOE derived from various cell types on Aβ fibrillization are unknown, the current data suggest that APOE particles secreted by microglia are smaller (and hence less lipidated) than those from astrocytes [53]. We currently do not know the molecular properties of APOE secreted by vascular mural cells and their exact contributions to CAA, although APOE4 pericytes may play an important role in disease progression [37]. Regardless, these findings suggest that APOE from distinct cellular sources may have different Aβ seeding potential. Interestingly, other studies in genetically modified mice have shown that altering microglial function into a less phagocytic state [73] or depleting microglia either pharmacologically [74] or genetically [75] – all of which likely decreases microglial-secreted APOE – increases the amount of CAA. To what degree APOE is altered has not been determined, although one study in a mouse model of tauopathy detected a compensatory mechanism where there was increased APOE protein in astrocytes and neurons after pharmacologically ablating microglia [76]. Furthermore, loss of clusterin (APOJ), another highly expressed apolipoprotein in the brain that deposits in parenchymal plaques and CAA [77], increased CAA deposition [78]. It is possible that regardless of the cellular source of APOE, an unbiased reduction of CNS APOE may lead to less aggregation of Aβ and hence less retention of Aβ in the brain parenchyma. Soluble Aβ produced in the brain and released into the brain ISF is cleared in part through the perivascular pathway, a likely source of Aβ that deposits as CAA. According to a related study, astrocytic APOE removal did not affect APP cleavage and production [48], suggesting that the shift in Aβ localization was not a result of altered Aβ production. Although we detected a two-fold increase in Aβ_40_ in penetrating vessels, whether this is a result of enhanced perivascular clearance is unknown. Therefore, determining whether ISF and perivascular Aβ clearance is altered in these mouse models including ours with loss of astrocytic APOE4 would be of future interest. Future experiments in which APOE4 is selectively removed from various CNS cell types could provide valuable insight into the role of cell-specific APOE in plaque/CAA pathogenesis.

In our current study, perhaps our most surprising finding was that astrocytic APOE4 removal led to increased CAA but was protective for the cerebrovasculature. Astrocytes have critical basal functions, including providing neurotrophic support, regulating ion homeostasis, and creating a physical barrier from the perivascular space [79, 80]. Along the cerebrovasculature, perivascular astrocytic end-feet occupy a barrier that allows for the flux of selective molecules [80]. However, under pathological conditions such as CAA or with an inflammatory challenge [81], homeostatic perivascular astrocytes can adopt a neurotoxic phenotype [42] and may have aberrant basal function such as the inability to regulate water channels (aquaporin 4) important for solute transport [46, 82]. Under pathological conditions, APOE is upregulated in astrocytes, termed disease-associated astrocytes (DAA) [41]. Removal of APOE4 from a mouse model of amyloidosis reduces GFAP reactivity in male mice [48], while single-nuclei RNA sequencing in a mouse model of tauopathy has revealed that numerous DAA genes such as *Clu* and *Vim* are reduced [49]. In our study, we also detected a reduction of select DAA genes including *C3*and *Gfap*, suggesting that APOE4 removal may revert pathological astrocytes to a more homeostatic state that in some way allows for protection and support for the cerebrovasculature. From our astrocyte morphological analysis, there were no overt changes in mice with or without astrocytic APOE4 depletion that would indicate the state of these astrocytes; however, GFAP^+^ astrocytes seemingly respond differently to plaques versus CAA. Further studies focusing on BBB-associated astrocytes are necessary to understand their role in a disease context and whether this state is transient and/or reversible.

APOE therapy has been a recent topic of interest as a novel strategy to target multiple neurodegenerative diseases including AD and CAA. Several strategies including APOE immunotherapy [54, 55, 83], antisense oligonucleotides [84], and APOE-mimetic peptides [85, 86] among others have shown preclinical promise for the treatment of amyloidosis without overt adverse effects on the cerebrovasculature or peripheral lipid homeostasis. Most of these studies, however, were not conducted in mice with CAA, which is a neuropathological feature detected in virtually all AD patients. This current study identified that in a mouse model of mixed CAA/plaque pathology, removal of a major source of APOE produced by astrocytes increased CAA but provided protection by reducing neuritic dystrophy and ameliorating CAA-induced vascular damage. APOE4 and CAA have both been implicated to exacerbate damage in the AD brain [4], and future studies investigating APOE should incorporate the usage and analysis of CAA in addition to Aβ plaques to more accurately model human Aβ pathology. Recently, our group generated an antibody that selectively targets a form of non-lipidated APOE4 only found in amyloid plaques and CAA [54] and compared its effects directly against an Aβ-targeting antibody. When the Aβ-targeting antibody was administered to 5XFAD/APOE4 mice that develop CAA and plaques, it did not decrease CAA and resulted in an increase in GFAP^+^ astrocytes lining CAA that strongly correlated with microhemorrhages, a major adverse effect in response to certain Aβ immunotherapy [54]. In contrast, the anti-APOE antibody decreased CAA, improved vascular function, and did not result in an increase in microhemorrhages or in GFAP^+^ astrocytes lining CAA [54]. Considering our current findings, it would be especially relevant to the current challenges of Aβ immunotherapy to determine whether modifying these GFAP^+^ astrocytes either genetically or pharmacologically would provide neuro/vascular protection and attenuate Aβ antibody-mediated vascular adverse effects. Given that vascular adverse effects (amyloid-related imaging abnormalities, ARIA) are a major setback to many Aβ-targeting antibodies, this is a critical question to resolve.

## Conclusion

APOE4 and CAA are both major risk factors for vascular dysfunction [87] by shared and disparate mechanisms. Our study has uncovered that attenuating APOE4 pathology by reducing astrocytic APOE4 production is sufficient to provide protection for CAA-associated vascular damage, including ameliorating BBB leakiness and improving vasoreactivity. Removal of APOE4 may revert astrocytes, including CAA-associated astrocytes, from a pathological to a physiological state that is neuro- and vascular-protective. These results suggest that there is therapeutic potential in utilizing APOE-targeting strategies for the treatment of CAA and AD, but more follow-up studies are necessary to uncouple the complex relationship between cell-specific APOE contributions to CAA development and vascular function.

## Supplementary Information

**Additional file 1: Figure S1.** No sex differences in APOE4 mRNA expression and protein concentrations in mice with or without Cre expression. a, Relative expression of *APOE* mRNA normalized to beta-actin in cortex. b, c, PBS-soluble and guanidine-HCL-soluble (“insoluble”) APOE protein concentrations assessed by ELISA from cortex. Data expressed as mean ± SD, two-way ANOVA, Sidak’s multiple comparisons test performed for all statistical analyses. No statistical comparisons are significant unless indicated.

**Additional file 2: Figure S2.** No sex differences in amyloid or Aβ pathology in mice with or without Cre expression. a–c, X34 staining for fibrillar plaques/CAA with percent area coverage of total X34 (a), CAA (b), and amyloid plaques (c) in the cortex overlying the hippocampus of 10-month-old 5X+AL- or 5X+AL+ mice after astrocytic APOE4 removal at 2-months-of-age. d–f, Aβ immunoreactivity (Aβ-IR) with percent area coverage of total Aβ-IR (d), vascular Aβ-IR (e), and plaques (f) in the cortex overlying the hippocampus. Data expressed as mean ± SD, two-way ANOVA, Sidak’s multiple comparisons test performed for all statistical analyses. No statistical comparisons are significant unless indicated.

**Additional file 3: Figure S3.** Increased CAA and reduction of amyloid plaques in the subiculum albeit no change in overall amyloid. a–d, X34 staining for fibrillar plaques/CAA (a) with percent area coverage of total X34 (b), CAA (c), and amyloid plaques (d) in the dorsal subiculum of 10-month-old 5X+AL- or 5X+AL + mice after astrocytic APOE4 removal at 2-months-of-age. e, Proportion of CAA in total X34^+^ staining. Scalebar: 300 µm. ∆ = males, ○ = females. Data expressed as mean ± SD, student’s *t*-test (two-sided) performed for all statistical analyses except (b), where Welch’s *t*-test was performed. **P < 0.01, ***P < 0.001. No other statistical comparisons are significant unless indicated.

**Additional file 4: Figure S4.** Tamoxifen-induced reduction of astrocytic APOE4 in cortical regions with and without CAA/plaques. a, Representative images of X34 for amyloid plaques/CAA, APOE, GFAP^+^ astrocytes, and IBA1^+^ microglia. Arrowhead: APOE in astrocyte. Arrow: APOE in microglia. Scalebar: 50 µm. b, Percentage of APOE coverage in cortical regions with CAA, plaques, or without CAA/plaques. c, d, Ratio of APOE in GFAP^+^ astrocytes (c) or IBA1^+^ (d) microglia area coverage normalized to CAA/plaque load. ∆ = males, ○ = females. Data expressed as mean ± SEM, student’s *t*-test (two-sided) performed for all statistical analyses except (b – no plaques, CAA, plaques), (c – no plaques, CAA, plaques), and (d – no plaques, CAA), where Welch’s *t*-test was performed. *P < 0.05, **P < 0.01. No other statistical comparisons are significant unless indicated.

**Additional file 5: Figure S5.** No change in GFAP^+^ astrocyte coverage in the subiculum. a, b, GFAP staining for astrocytes (a) with percent area coverage of total GFAP immunoreactivity (b) in the dorsal subiculum of 10-month-old 5X+AL- or 5X+AL + mice after astrocytic APOE4 removal at 2-months-of-age. Scalebar: 300 µm. ∆ = males, ○ = females. Data expressed as mean ± SD, student’s *t*-test (two-sided) performed for all statistical analyses. No statistical comparisons are significant unless indicated.

**Additional file 6: Figure S6.** Characterization of morphological responses of GFAP^+^ astrocytes to CAA or plaques. a, Representative reconstruction of GFAP^+^ astrocytes around CAA or amyloid plaques using Simple Neurite Tracer. b–f, Morphological analyses of GFAP + astrocyte size (Convex Hull analysis) (b), number of processes (c), total processes length (d), number of branching points (e), and mean process length (f). Scalebar: 50 µm. ∆ = males, ○ = females. Data expressed as mean ± SD, two-way ANOVA, Sidak’s multiple comparisons test performed for all statistical analyses. *P < 0.05, **P < 0.01. ***P < 0.001. No other statistical comparisons are significant unless indicated.

## Data Availability

Generated datasets used for analyses in this study are available from the corresponding author upon reasonable request.

## Abbreviations

5XFAD: 5 Familial AD mutations
Aβ: Amyloid-β
AD: Alzheimer disease
APP: Amyloid precursor protein
APOE: Apolipoprotein E
APOJ: Apolipoprotein J
ARIA: Amyloid-related imaging abnormalities
CAA: Cerebral amyloid angiopathy
CNS: Central nervous system
CSF: Cerebrospinal fluid
DAA: Disease-associated astrocytes
ELISA: Enzyme-linked immunosorbent assay
GFAP: Glial fibrillary acidic protein
IBA1: Ionized calcium binding adaptor molecule 1
ISF: Interstitial fluid
i.p.: Intraperitoneal
LAMP1: Lysosomal-associated membrane protein 1
qPCR: Quantitative polymerase chain reaction
SMC: Smooth muscle cell
SNAP: *S*-Nitroso-*N*-acetylpenicillamine
TBS: Tris-buffered saline
