# Supplementary figures and images for: Astrocytic APOE4 removal confers cerebrovascular protection despite increased cerebral amyloid angiopathy
Source: Mol Neurodegener. 2023 Mar 16;18:17. doi: 10.1186/s13024-023-00610-x (PMC10018855; doi:10.1186/s13024-023-00610-x)

Figure S1

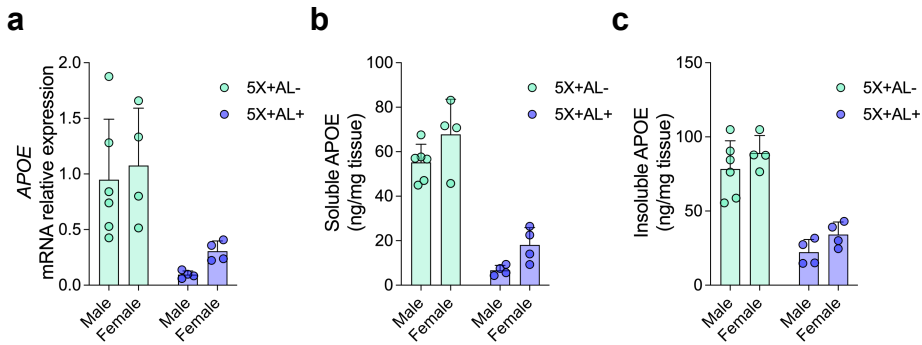

Supplement: Supplementary file 1 — Additional file 1: Figure S1. No sex differences in APOE4 mRNA expression and protein concentrations in mice with or without Cre expression. a, Relative expression of APOE mRNA normalized to beta-actin in cortex. b, c, PBS-soluble and guanidine-HCL-soluble (“insoluble”) APOE protein concentrations assessed by ELISA from cortex. Data expressed as mean ± SD, two-way ANOVA, Sidak’s multiple comparisons test performed for all statistical analyses. No statistical comparisons are significant unless indicated. [file 13024_2023_610_MOESM1_ESM.pdf]

Figure S2

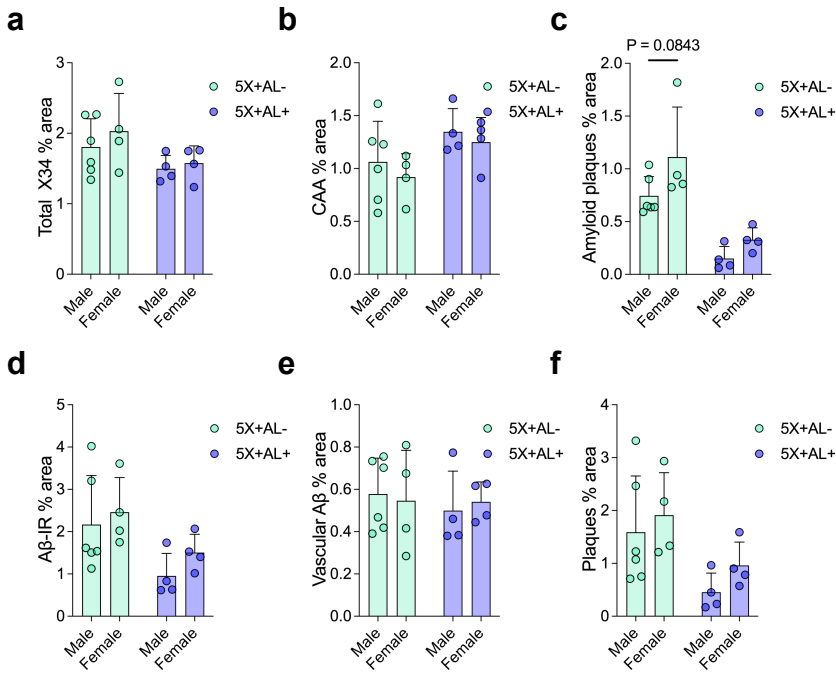

Supplement: Supplementary file 2 — Additional file 2: Figure S2. No sex differences in amyloid or Aβ pathology in mice with or without Cre expression. a–c, X34 staining for fibrillar plaques/CAA with percent area coverage of total X34 (a), CAA (b), and amyloid plaques (c) in the cortex overlying the hippocampus of 10-month-old 5X+AL- or 5X+AL+ mice after astrocytic APOE4 removal at 2-months-of-age. d–f, Aβ immunoreactivity (Aβ-IR) with percent area coverage of total Aβ-IR (d), vascular Aβ-IR (e), and plaques (f) in the cortex overlying the hippocampus. Data expressed as mean ± SD, two-way ANOVA, Sidak’s multiple comparisons test performed for all statistical analyses. No statistical comparisons are significant unless indicated. [file 13024_2023_610_MOESM2_ESM.pdf]

Figure S3

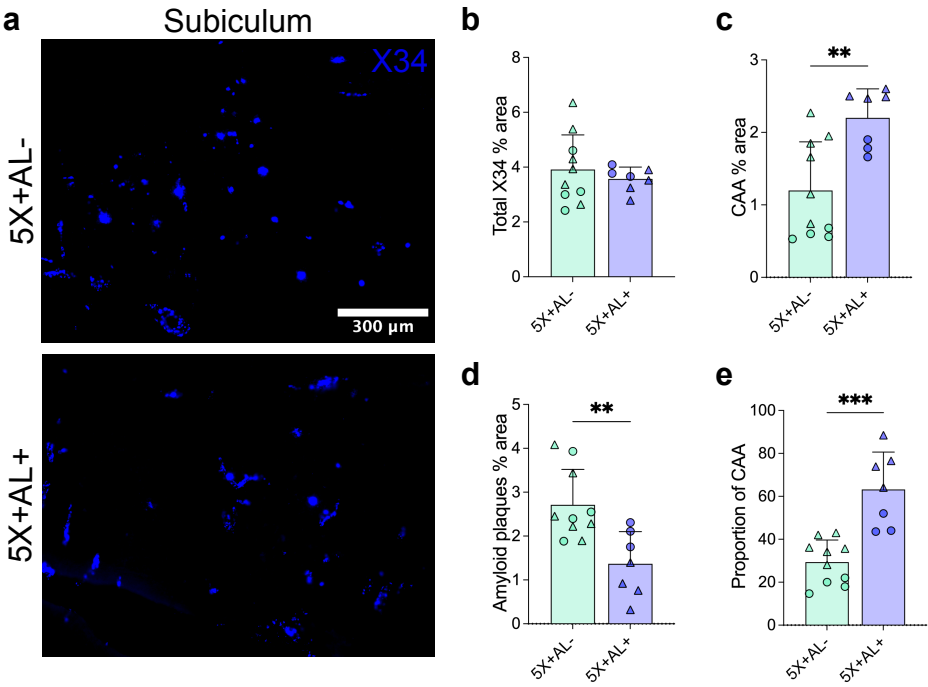

Supplement: Supplementary file 3 — Additional file 3: Figure S3. Increased CAA and reduction of amyloid plaques in the subiculum albeit no change in overall amyloid. a–d, X34 staining for fibrillar plaques/CAA (a) with percent area coverage of total X34 (b), CAA (c), and amyloid plaques (d) in the dorsal subiculum of 10-month-old 5X+AL- or 5X+AL + mice after astrocytic APOE4 removal at 2-months-of-age. e, Proportion of CAA in total X34+ staining. Scalebar: 300 µm. ∆ = males, ○ = females. Data expressed as mean ± SD, student’s t-test (two-sided) performed for all statistical analyses except (b), where Welch’s t-test was performed. **P < 0.01, ***P < 0.001. No other statistical comparisons are significant unless indicated. [file 13024_2023_610_MOESM3_ESM.pdf]

Figure S4

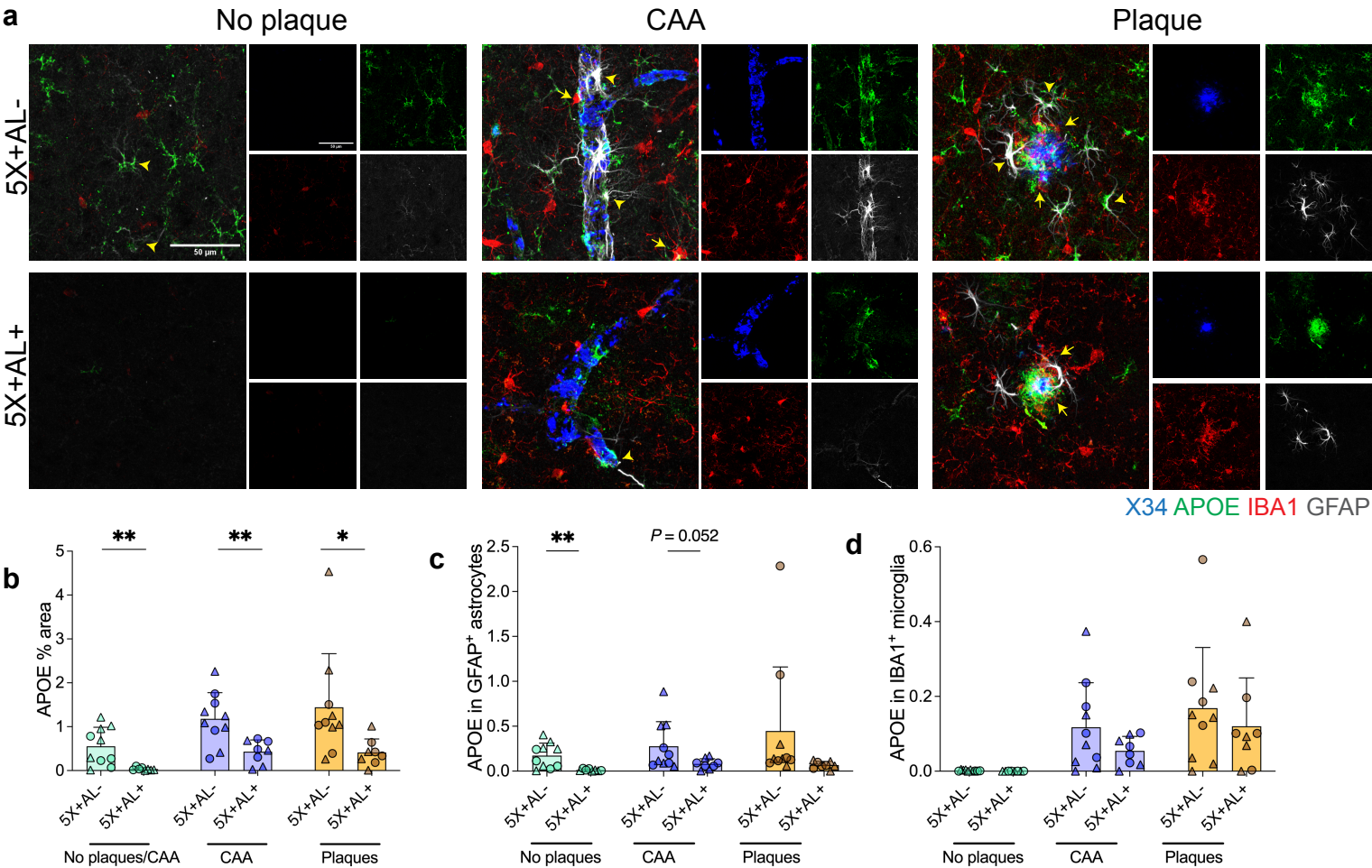

Supplement: Supplementary file 4 — Additional file 4: Figure S4. Tamoxifen-induced reduction of astrocytic APOE4 in cortical regions with and without CAA/plaques. a, Representative images of X34 for amyloid plaques/CAA, APOE, GFAP+ astrocytes, and IBA1+ microglia. Arrowhead: APOE in astrocyte. Arrow: APOE in microglia. Scalebar: 50 µm. b, Percentage of APOE coverage in cortical regions with CAA, plaques, or without CAA/plaques. c, d, Ratio of APOE in GFAP+ astrocytes (c) or IBA1+ (d) microglia area coverage normalized to CAA/plaque load. ∆ = males, ○ = females. Data expressed as mean ± SEM, student’s t-test (two-sided) performed for all statistical analyses except (b – no plaques, CAA, plaques), (c – no plaques, CAA, plaques), and (d – no plaques, CAA), where Welch’s t-test was performed. *P < 0.05, **P < 0.01. No other statistical comparisons are significant unless indicated. [file 13024_2023_610_MOESM4_ESM.pdf]

Figure S5

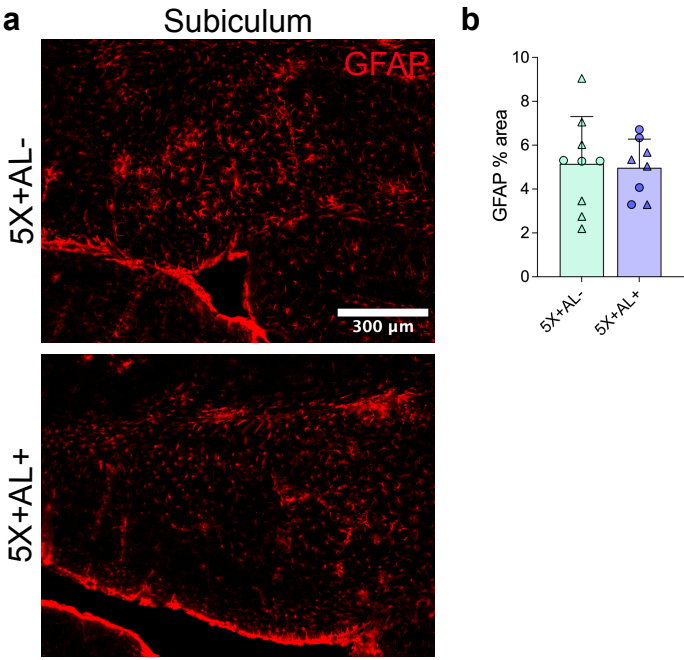

Supplement: Supplementary file 5 — Additional file 5: Figure S5. No change in GFAP+ astrocyte coverage in the subiculum. a, b, GFAP staining for astrocytes (a) with percent area coverage of total GFAP immunoreactivity (b) in the dorsal subiculum of 10-month-old 5X+AL- or 5X+AL + mice after astrocytic APOE4 removal at 2-months-of-age. Scalebar: 300 µm. ∆ = males, ○ = females. Data expressed as mean ± SD, student’s t-test (two-sided) performed for all statistical analyses. No statistical comparisons are significant unless indicated. [file 13024_2023_610_MOESM5_ESM.pdf]

Figure S6

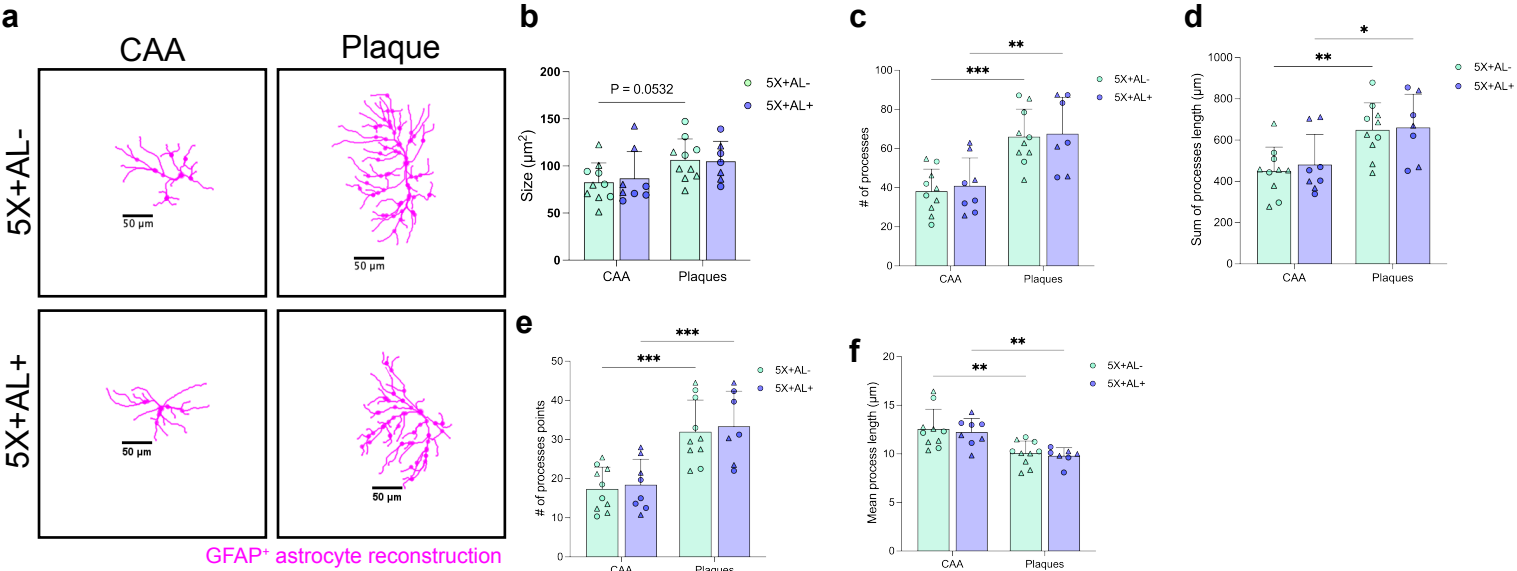

Supplement: Supplementary file 6 — Additional file 6: Figure S6. Characterization of morphological responses of GFAP+ astrocytes to CAA or plaques. a, Representative reconstruction of GFAP+ astrocytes around CAA or amyloid plaques using Simple Neurite Tracer. b–f, Morphological analyses of GFAP + astrocyte size (Convex Hull analysis) (b), number of processes (c), total processes length (d), number of branching points (e), and mean process length (f). Scalebar: 50 µm. ∆ = males, ○ = females. Data expressed as mean ± SD, two-way ANOVA, Sidak’s multiple comparisons test performed for all statistical analyses. *P < 0.05, **P < 0.01. ***P < 0.001. No other statistical comparisons are significant unless indicated. [file 13024_2023_610_MOESM6_ESM.pdf]
